# T2b Gallbladder Adenocarcinoma Diagnosed by Frozen Section With High-Grade Dysplasia at the Cystic Duct Margin: Diagnostic and Prognostic Considerations

**DOI:** 10.7759/cureus.109698

**Published:** 2026-05-26

**Authors:** Christopher M Ahmad, Rachana Tadakamalla, Rae-Anne Kastle, Salman Muqeet, Amer Abboud

**Affiliations:** 1 Internal Medicine, Kansas City University, Joplin, USA; 2 Pathology, Kansas City University, Joplin, USA; 3 Pathology and Laboratory Medicine, Humboldt Park Health, Chicago, USA

**Keywords:** allbladder adenocarcinoma, high-grade dysplasia, t2b disease, tubulovillous adenoma, tumor invasion depth

## Abstract

Gallbladder adenocarcinoma is an uncommon gastrointestinal malignancy that is frequently discovered incidentally during cholecystectomy. Intraoperative frozen section is often used to guide surgical decision-making but has inherent limitations, particularly in assessing depth of invasion. We report the case of a 78-year-old female who underwent an elective laparoscopic cholecystectomy for a gallbladder mass. Intraoperative frozen section demonstrated a tubulovillous adenoma with at least carcinoma in situ. Subsequent permanent histologic evaluation revealed a moderately differentiated adenocarcinoma invading the hepatic-side perimuscular connective tissue, consistent with pT2b disease. Despite the advanced T stage, the tumor exhibited several relatively favorable histologic features, including negative margins and absence of lymphovascular invasion, although nodal assessment was limited. The cystic duct margin showed focal high-grade dysplasia without invasive carcinoma. This case highlights the diagnostic limitations of frozen-section analysis in gallbladder pathology and underscores the importance of integrating final histologic findings when assessing prognosis and postoperative management in T2b disease.

## Introduction

Gallbladder carcinoma is a rare malignancy, often diagnosed incidentally following cholecystectomy performed for presumed benign disease [1]. Accurate pathologic staging is critical, as depth of invasion remains one of the most important prognostic factors [2,3]. Intraoperative frozen section is frequently employed to provide rapid diagnostic information when a suspicious lesion is encountered, potentially influencing the extent of surgery. However, frozen-section interpretation in biliary tract pathology is limited by sampling constraints, tissue artifact, and difficulty assessing invasion of the gallbladder wall [4]. Intraoperative diagnostic interpretation has immediate surgical implications, as identification of invasive carcinoma may prompt conversion from simple cholecystectomy to radical cholecystectomy, which typically includes resection of hepatic segments IVb and V along with regional lymphadenectomy [5]. Failure to detect invasion intraoperatively may therefore delay definitive oncologic resection.

Tumors invading the perimuscular connective tissue are classified as T2 disease, with hepatic-side invasion (T2b) generally associated with worse outcomes than peritoneal-side invasion (T2a) [2,3]. The hepatic side of the gallbladder is directly contiguous with the liver, lacks a serosal barrier, and exhibits distinct lymphatic and vascular drainage patterns, facilitating early local spread and contributing to poorer survival and higher recurrence in T2b disease compared to T2a tumors. This anatomic relationship underscores the importance of accurately identifying the depth and location of invasion during pathologic assessment. Reported long-term outcomes for T2 gallbladder carcinoma vary substantially by tumor location and associated histologic risk factors, underscoring the importance of accurate staging and complete pathologic assessment. Nevertheless, prognosis within T2b disease is heterogeneous and influenced by additional histologic factors such as margin status, lymphovascular invasion, and nodal involvement [2,3]. This case illustrates both the diagnostic limitations of frozen-section analysis and the prognostic nuances of T2b gallbladder adenocarcinoma lacking aggressive histologic features.

Gallbladder carcinogenesis is often thought to follow an adenoma-dysplasia-carcinoma sequence, in which progressive epithelial atypia culminates in invasive malignancy [6]. This stepwise progression helps explain why limited sampling during frozen-section analysis may capture dysplasia or carcinoma in situ while missing deeper invasive components.

## Case presentation

A 78-year-old woman presented for evaluation following the incidental detection of a gallbladder mass on imaging during workup for nonspecific abdominal discomfort. The patient denied significant constitutional symptoms, including weight loss, jaundice, or persistent abdominal pain. Preoperative imaging demonstrated a focal gallbladder lesion with irregular morphology, raising concern for neoplastic transformation and prompting surgical management.

Radiologic evaluation of gallbladder lesions can be challenging, as benign polyps, adenomas, and early malignancies often demonstrate overlapping imaging features. Small polypoid lesions are frequently interpreted as benign, particularly when detected incidentally; however, lesions exceeding several millimeters in size or demonstrating irregular or lobulated morphology typically warrant surgical removal due to malignant potential. The patient subsequently underwent an elective laparoscopic cholecystectomy at Humboldt Park Health in Chicago, Illinois. No additional relevant clinical history was identified.

Given the lesion’s size (2.5 cm), irregular lobulated morphology, and associated focal wall thickening, the findings exceeded typical thresholds for benign gallbladder polyps and raised concern for neoplastic transformation. These features prompted an elective cholecystectomy for definitive diagnosis and management.

An intact gallbladder measuring 7 × 3 × 2 cm was received fresh in the pathology department for intraoperative consultation. The serosal surface was pink and smooth. On opening, a pink, lobulated, polypoid mass measuring 2.5 × 1.5 × 0.5 cm was identified in the fundus (Table 1). On gross examination, the gallbladder wall appeared mildly thickened in the region surrounding the mass. The mucosal surface overlying the lesion demonstrated focal irregularity with a lobulated architecture. The mass itself had a papillary and villiform surface configuration, suggestive of an underlying adenomatous lesion. Sectioning revealed a tan-pink, friable lesion projecting into the lumen with focal areas of induration within the underlying wall. The adjacent mucosa appeared unremarkable without additional discrete lesions. No gallstones were identified within the lumen. The cystic duct margin appeared grossly unremarkable and was submitted for histologic evaluation. Features such as lesion size, irregular morphology, and focal wall thickening raised concern for neoplastic transformation rather than a benign polyp.

**Table 1:** Key pathologic findings and staging features. AJCC: American Joint Committee on Cancer.

Feature	Finding
Tumor size	2.5 × 1.5 × 0.5 cm
Location	Fundus, hepatic side
Frozen section diagnosis	Tubulovillous adenoma with carcinoma in situ
Final diagnosis	Moderately differentiated adenocarcinoma (Grade II/III)
Stage	pT2bN0M0, Stage IIB (AJCC 8th Edition)
Margins	Negative (0.5 cm to inked edge)
Lymphovascular invasion	Not seen
Nodal status	1 node examined, negative
Cystic duct margin	High-grade dysplasia, no invasive carcinoma

The specimen was inked, serially sectioned, and representative sections of the mass were submitted for frozen section evaluation. Intraoperative frozen section demonstrated a tubulovillous adenoma with at least carcinoma in situ. Given the limitations of frozen-section assessment of invasion, complete submission of the tumor for permanent histologic evaluation was recommended.

**Figure 1:**
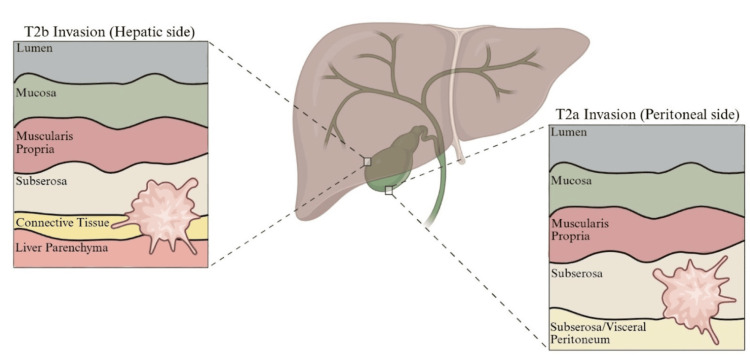
Schematic diagram of gallbladder wall layers illustrating T2a versus T2b invasion. This is an original author-created schematic illustrating the anatomical layers of the gallbladder wall and the distinction between peritoneal-side (T2a) and hepatic-side (T2b) tumor invasion. The image was created using BioRender. Image Credit: Rae-Anne Kastle.

Following formalin fixation, permanent sections revealed a moderately differentiated adenocarcinoma (Grade II/III) infiltrating the perimuscular connective tissue on the hepatic side (T2b) without extension into the liver parenchyma (Figure 1). The inked surgical margin was negative for tumor, with the closest margin measuring 0.5 cm. The tumor was located 0.7 cm from the liver parenchyma. One lymph node was examined and was negative for metastatic disease. No lymphovascular invasion was identified. The cystic duct margin demonstrated focal high-grade dysplasia without invasive carcinoma (Table 1).

**Figure 2:**
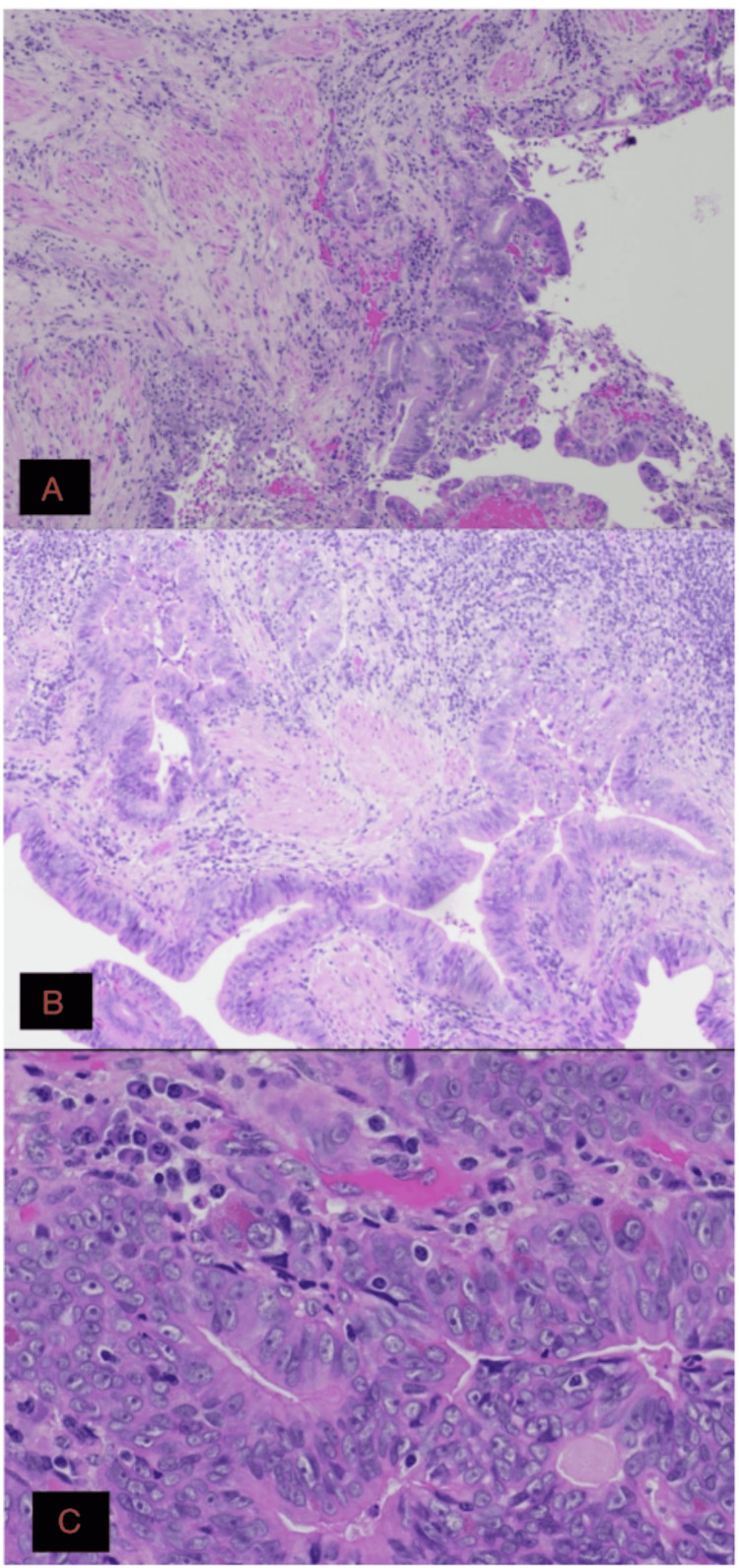
Hematoxylin and eosin–stained section demonstrating T2b invasive adenocarcinoma. (A) Low-power hematoxylin and eosin–stained section (10×) demonstrating irregular, angulated glandular structures infiltrating a desmoplastic fibromuscular stroma on the hepatic side, consistent with invasive adenocarcinoma. (B) Intermediate-power hematoxylin and eosin–stained section showing irregular, angulated tumor glands infiltrating desmoplastic fibrous stroma, confirming invasive adenocarcinoma beyond the epithelial surface. (C) High-power hematoxylin and eosin–stained section demonstrating irregular tumor glands infiltrating the fibrous tissue, confirming invasive adenocarcinoma.

Specimen fixation in 10% neutral buffered formalin was initiated within 20 minutes of resection and continued for more than six hours but less than 72 hours. Final pathologic staging was pT2bN0M0, corresponding to Stage IIB disease according to the American Joint Committee on Cancer (AJCC) Eighth Edition (Table 1). Microscopically, the tumor was composed of irregularly shaped and angulated glandular structures infiltrating the perimuscular connective tissue. The glands were lined by atypical columnar epithelial cells exhibiting enlarged hyperchromatic nuclei, nuclear stratification, and moderate cytologic atypia. Occasional nucleoli were present. The tumor glands were embedded within a desmoplastic stromal response characterized by dense fibrous tissue and scattered inflammatory cells. Mitotic figures were present but not numerous. Areas of residual adenomatous epithelium with villous architecture were identified adjacent to invasive tumor glands, supporting the presence of an adenoma-carcinoma sequence. Microscopic findings are shown in Figure 2.

At early postoperative follow-up, the patient remained clinically stable, without evidence of immediate postoperative complications or documented disease progression. Long-term surveillance planning was initiated, although extended follow-up data were not available at the time of this report. At early postoperative follow-up, the patient remained clinically stable without complications. Ongoing management included plans for multidisciplinary evaluation and structured surveillance, although long-term follow-up data were not available at the time of this report.

## Discussion

This case highlights two clinically relevant themes: the diagnostic limitations of intraoperative frozen-section analysis in gallbladder neoplasia and the prognostic complexity of T2b gallbladder adenocarcinoma in the absence of adverse histologic features, as shown in Figures 2A-C.

Diagnostic limitations of frozen section

Frozen section evaluation was requested intraoperatively due to concern for neoplasia based on the gross appearance of the lesion. Frozen section is a valuable intraoperative tool but is inherently limited in its ability to assess depth of invasion, particularly in gallbladder specimens, where the wall architecture is thin, and sampling is necessarily restricted [7]. During intraoperative consultation, the specimen was rapidly sectioned, and representative portions of the lesion were embedded in optimal cutting temperature compound for cryostat sectioning. Frozen-section preparation allows rapid histologic evaluation but frequently introduces freezing artifacts that may obscure architectural details. In addition, only limited tissue can be examined during intraoperative consultation, which may not fully represent the invasive component of a lesion. These technical factors are particularly relevant in gallbladder pathology, where the thin wall and complex architecture can make identification of subtle invasion difficult on frozen sections. In this case, the frozen section correctly identified carcinoma in situ but failed to detect invasion into the hepatic-side perimuscular connective tissue. The differences between the intraoperative frozen-section interpretation and the final permanent histologic findings are summarized in Table 2.

**Table 2:** Comparison of intraoperative frozen section and final permanent histology findings. This table summarizes the differences between intraoperative frozen-section interpretation and final permanent histologic evaluation in this patient. The table presents original content created by the authors based on findings from the present case.

Feature	Frozen Section	Permanent Histology
Epithelial diagnosis	Tubulovillous adenoma with carcinoma in situ	Moderately differentiated adenocarcinoma
Depth of invasion	Not assessed	Invasion into hepatic-side perimuscular connective tissue (T2b)
Surgical margins	Not assessed	Negative (0.5 cm clearance)
Lymphovascular invasion	Not assessed	Not identified
Nodal status	Not assessed	One lymph node examined, negative

This underestimation likely reflects sampling limitations rather than interpretive error. The case reinforces the importance of cautious intraoperative decision-making and complete tumor submission for permanent histologic evaluation when frozen section suggests high-grade dysplasia or carcinoma in situ. The presence of a tubulovillous adenoma adjacent to invasive adenocarcinoma suggests progression along an adenoma-carcinoma sequence similar to that observed in other gastrointestinal malignancies. In this pathway, dysplastic epithelial proliferation within an adenomatous lesion gradually accumulates genetic and structural abnormalities that ultimately lead to invasive carcinoma. Identification of residual adenomatous architecture adjacent to invasive tumor glands supports this progression and provides insight into the pathogenesis of the malignancy in this case.

Prognostic considerations in T2b disease

Hepatic-side perimuscular invasion has generally been associated with higher recurrence rates and poorer survival compared to peritoneal-side tumors [8]. However, T stage alone does not fully capture biological behavior. In this patient, several favorable histologic features were present, including negative surgical margins, absence of lymphovascular invasion, and node-negative disease. The key prognostic features identified in this case are summarized in Table 3.

**Table 3:** Prognostic histologic factors relevant to T2b gallbladder adenocarcinoma in this case.

Prognostic Factor	Finding in This Case	Clinical Significance
Tumor stage	pT2b	Hepatic-side invasion associated with higher recurrence risk
Tumor differentiation	Moderately differentiated	Intermediate biologic aggressiveness
Surgical margins	Negative (0.5 cm clearance)	Favorable prognostic factor
Lymphovascular invasion	Not identified	Reduced risk of metastatic spread
Nodal status	1 node examined, negative	Favorable prognostic indicator
Adjacent lesion	Tubulovillous adenoma	Supports adenoma–carcinoma progression
Cystic duct margin	High-grade dysplasia only	May warrant surveillance but not residual invasive disease

These factors are known to influence prognosis and may mitigate the adverse implications typically associated with T2b disease [9]. The presence of focal high-grade dysplasia at the cystic duct margin did not alter pathologic staging and was not associated with invasive carcinoma. The finding of high-grade dysplasia at the cystic duct margin raises important diagnostic considerations. Dysplasia within the biliary epithelium may represent either a precursor lesion or part of a broader field change affecting the biliary tract mucosa. Although dysplasia does not constitute invasive carcinoma, its presence indicates significant epithelial atypia and may reflect a biologic environment conducive to malignant transformation. 

Consequently, the identification of dysplasia at a surgical margin may influence postoperative surveillance strategies even when invasive carcinoma is absent at the margin. While the clinical significance of dysplasia at the cystic duct margin remains uncertain, its identification may justify careful postoperative surveillance [10]. Although high-grade dysplasia at the cystic duct margin does not constitute invasive disease, its presence may raise concern for field effect or synchronous epithelial atypia, warranting close clinical follow-up. A key limitation in this case is the evaluation of only a single lymph node. Current AJCC recommendations suggest assessing at least six lymph nodes for accurate nodal staging in gallbladder carcinoma [11]. Therefore, classification as N0 in this case should be interpreted with caution, as occult micrometastatic disease cannot be definitively excluded.

Management implications

Intraoperative management of suspected gallbladder neoplasia presents a challenging clinical scenario. Surgeons must often decide whether to proceed with extended resection based on limited intraoperative information. Frozen-section analysis may identify dysplasia or carcinoma in situ but may not reliably demonstrate invasion into deeper layers of the gallbladder wall. As a result, the surgeon must weigh the potential benefits of immediate radical surgery against the risks associated with more extensive operative intervention. This case underscores the importance of integrating final histologic findings into postoperative decision-making. Although extended resection is often considered for T2b tumors, the absence of high-risk histologic features in this case supports an individualized approach rather than relying solely on T stage [12]. A multidisciplinary discussion remains essential when frozen-section findings and final pathology diverge. Current recommendations for extended resection in T2b disease vary across institutions and guidelines, underscoring the importance of individualized decision-making.

Beyond traditional staging metrics, emerging data suggest that gallbladder adenocarcinoma demonstrates significant biologic heterogeneity even within identical T classifications [13]. Molecular profiling studies have identified variability in KRAS mutations, TP53 alterations, HER2 overexpression, and mismatch repair status, all of which may influence tumor behavior and recurrence risk [14]. Although molecular testing is not yet routinely incorporated into staging algorithms for gallbladder carcinoma, these findings highlight the limitations of relying solely on anatomic invasion patterns to predict outcomes. In a patient such as ours, classified as T2b but lacking lymphovascular invasion, nodal metastasis, or margin positivity, the tumor’s biologic aggressiveness may differ substantially from population-level survival estimates traditionally associated with hepatic-side invasion [15]. Future integration of molecular and histologic risk stratification may allow more precise postoperative management strategies.

Another consideration in this case involves the surgical decision-making framework when frozen-section findings underestimate the final pathologic stage. Intraoperative decisions are often made under time constraints and based on incomplete sampling, particularly in biliary tract malignancies, where subtle invasion may not be evident in limited sections [16]. When a frozen section demonstrates carcinoma in situ or high-grade dysplasia, the surgeon must balance the risk of undertreatment against the morbidity of immediate extended resection [8]. Retrospective analyses suggest that re-resection after incidental discovery of gallbladder cancer can improve survival in appropriately selected T2 patients. However, the magnitude of benefit may vary with nodal status, margin involvement, and biologic factors [17]. This case underscores the importance of structured postoperative reassessment when final pathology reveals a higher stage than initially suspected.

The presence of high-grade dysplasia at the cystic duct margin also warrants further reflection. While dysplasia alone does not upstage disease or constitute residual invasive carcinoma, it may represent either a field effect within the biliary epithelium or a precursor lesion with potential for malignant transformation [18]. The true prognostic significance of isolated dysplasia at the cystic duct margin remains unclear, and management strategies range from observation to consideration of additional resection in selected patients [18]. In the absence of invasive carcinoma at the margin, most contemporary approaches favor close surveillance rather than immediate further surgery [9]. However, this decision should be individualized based on patient comorbidities, operative risk, and multidisciplinary consensus. While not indicative of residual invasive disease, high-grade dysplasia at the cystic duct margin may reflect a field effect and supports the need for structured postoperative surveillance. In the absence of invasive carcinoma at the margin, this finding does not, in itself, warrant additional resection but does support the need for careful postoperative surveillance. Although the margin was negative, the relatively close proximity of the tumor to the liver parenchyma underscores the importance of considering local recurrence risk in T2b disease.

Postoperative management of T2b gallbladder carcinoma typically includes consideration of re-resection with hepatic segment IVb/V resection and regional lymphadenectomy when initial surgery is limited to simple cholecystectomy. In this case, a multidisciplinary evaluation is essential to determine the potential benefit of additional surgical intervention versus surveillance, particularly given favorable histologic features and patient-specific factors.

Referral for multidisciplinary evaluation, including surgical oncology and medical oncology, was considered to determine the potential role of re-resection and adjuvant therapy. Given the patient’s pathologic findings and overall clinical status, management decisions were individualized with consideration of operative risk and expected benefit.

Limitations

This case report is limited by its single-patient design, which restricts generalizability. Although the tumor demonstrated favorable histologic features, outcomes in T2b gallbladder adenocarcinoma are heterogeneous, and a single case cannot redefine expected prognostic patterns. Lymph node assessment was limited to a single node, potentially underestimating the likelihood of occult nodal disease and limiting the certainty of pathologic staging. Additionally, long-term follow-up data were not available at the time of publication, preventing assessment of recurrence or survival outcomes. Intraoperative frozen-section findings reflect the inherent sampling limitations of the technique, but this case does not allow quantification of diagnostic accuracy. Management decisions were individualized and may not be universally applicable across institutions. Another limitation relates to sampling inherent in surgical pathology evaluation. Although the tumor was completely submitted for histologic analysis, microscopic invasion patterns can occasionally be heterogeneous, and small foci of deeper invasion may be difficult to detect in limited sections. Additionally, lymph node evaluation was limited to a single node in this case, potentially underestimating the risk of microscopic nodal disease. Current AJCC recommendations suggest assessing at least six lymph nodes for accurate nodal staging in gallbladder carcinoma. Therefore, classification as N0 in this case should be interpreted with caution, as occult micrometastatic disease cannot be definitively excluded.

## Conclusions

The intraoperative frozen section plays an important role in evaluating gallbladder lesions but has limited ability to assess the depth of invasion. Permanent histologic evaluation remains essential for accurate staging. This case demonstrates that T2b gallbladder adenocarcinoma can present with relatively favorable histologic features; however, it does not establish a definitive management strategy and instead underscores the importance of individualized multidisciplinary decision-making. Careful correlation among intraoperative findings, permanent histology, and clinical context is therefore critical for guiding appropriate postoperative management. When a frozen section identifies high-grade dysplasia or carcinoma in situ in a suspicious gallbladder lesion, definitive prognostic assessment should be deferred until permanent histology is available. Management decisions for T2b gallbladder carcinoma should not be based solely on T stage. Rather, this case highlights the importance of individualized, multidisciplinary reassessment that incorporates final histopathology, extent of nodal evaluation, patient-specific factors, and operative risk when determining the need for additional surgical intervention. Interpretation of prognosis should therefore be made cautiously, given the limited lymph node assessment and absence of long-term follow-up data. Overall, this case highlights the potential discordance between pathologic staging and biologic behavior, emphasizing the need for integrated clinical, surgical, and histologic decision-making.
